# Mechanobiological Strategies to Enhance Ovine (*Ovis aries*) Adipose-Derived Stem Cells Tendon Plasticity for Regenerative Medicine and Tissue Engineering Applications

**DOI:** 10.3390/ani14152233

**Published:** 2024-07-31

**Authors:** Arlette A. Haidar-Montes, Annunziata Mauro, Mohammad El Khatib, Giuseppe Prencipe, Laura Pierdomenico, Umberto Tosi, Guy Wouters, Adrián Cerveró-Varona, Paolo Berardinelli, Valentina Russo, Barbara Barboni

**Affiliations:** 1 Unit of Basic and Applied Biosciences, Faculty of Bioscience and Agro-Food and Environmental Technology, University of Teramo, 64100 Teramo, Italy; aahaidarmontes@unite.it (A.A.H.-M.); melkhatib@unite.it (M.E.K.); gprencipe@unite.it (G.P.); utosi@unite.it (U.T.); acerverovarona@unite.it (A.C.-V.); pberardinelli@unite.it (P.B.); vrusso@unite.it (V.R.); bbarboni@unite.it (B.B.); 2 Center for Advanced Studies and Technology (CAST), University “G. d’Annunzio” of Chieti-Pescara, 66100 Chieti, Italy; laura.pierdomenico@unich.it; 3 FAT STEM Company, Erembodegem, 9300 Aalst, Belgium; guy.wouters@fat-stem.be

**Keywords:** adipose stem cells, ovine, mesenchymal stem cells, tendon, regenerative medicine, tissue engineering, conditioned media, scaffolds, tenogenic differentiation, signal transduction

## Abstract

**Simple Summary:**

Tendon injuries are known to be difficult to heal, making effective treatments crucial. This study examines the potential of stem cells derived from sheep fat tissue for tendon repair, since little information is available on their tenogenic potential. The focus of this study was to understand how these stem cells can be induced to develop into tendon cells. Initially, the cells were grown in the lab to ensure that their essential properties were maintained. Two methods were then employed to promote the acquisition of tendon-like characteristics: exposing the cells to a tenogenic conditioned medium or seeding them on a scaffold that mimics a tendon structure. The results indicated that these stem cells could be expanded without aging or changes in their characteristics. When exposed to the tendon medium, the cells were able to start the tendon differentiation process but were not able to complete it. Conversely, when seeded on the scaffold, the cells changed their naïve genetic and protein profile and acquired that of the typical tendon cells. This suggests that these stem cells can be addressed towards tendon cells, being more effective when seeded on a tendon biomimetic scaffold. These findings highlight the potential use of these stem cell treatments for tendon injuries, particularly in veterinary medicine.

**Abstract:**

Adipose-derived stem cells (ADSCs) hold promise for tendon repair, even if their tenogenic plasticity and underlying mechanisms remain only partially understood, particularly in cells derived from the ovine animal model. This study aimed to characterize oADSCs during in vitro expansion to validate their phenotypic properties pre-transplantation. Moreover, their tenogenic potential was assessed using two in vitro-validated approaches: (1) teno-inductive conditioned media (CM) derived from a co-culture between ovine amniotic stem cells and fetal tendon explants, and (2) short- (48 h) and long-term (14 days) seeding on highly aligned PLGA (ha-PLGA) electrospun scaffold. Our findings indicate that oADSCs can be expanded without senescence and can maintain the expression of stemness (Sox2, Oct4, Nanog) and mesenchymal (CD29, CD166, CD44, CD90) markers while remaining negative for hematopoietic (CD31, CD45) and MHC-II antigens. Of note, oADSCs’ tendon differentiation potential greatly depended on the in vitro strategy. oADSCs exposed to CM significantly upregulated tendon-related genes (COL1, TNMD, THBS4) but failed to accumulate TNMD protein at 14 days of culture. Conversely, oADSCs seeded on ha-PLGA fleeces quickly upregulated the tendon-related genes (48 h) and in 14 days accumulated high levels of the TNMD protein into the cytoplasm of ADSCs, displaying a tenocyte-like morphology. This mechano-sensing cellular response involved a complete SOX9 downregulation accompanied by YAP activation, highlighting the efficacy of biophysical stimuli in promoting tenogenic differentiation. These findings underscore oADSCs’ long-term self-renewal and tendon differentiative potential, thus opening their use in a preclinical setting to develop innovative stem cell-based and tissue engineering protocols for tendon regeneration, applied to the veterinary field.

## 1. Introduction

Tendons are critical components of the musculoskeletal system, linking muscles to bones and making movement possible. This specialized uniaxial connective tissue is characterized by an abundant and highly organized Extracellular Matrix (ECM). The matrix comprises tight packs of collagen type I (COL1) fibers oriented along the axis of the tendon, allowing force transmission [1]. The dynamic role of tendons makes them particularly prone to injury. Trauma, overuse, sports practice, or aging can increase the risk of pain-inducing disorders known as Tendinopathies. Unfortunately, adult tendons have poor spontaneous healing capacities due to low cellularization and hypovascularization. Despite their high incidence, resolutive treatment options for tendinopathies remain a challenge. The classic therapeutic approaches rely mainly on surgical or noninvasive techniques, often followed by a slow recovery, scar tissue formation, a reduction in biomechanical strength, and a high re-injury rate [2,3].

Regenerative medicine offers promising alternatives to enhance the effectiveness of treatments for tendon repair. This approach involves multiple considerations, including the choice of cell source, biomaterials, growth factors, mechanical stimuli, or their combinations [4,5,6]. They all aim to deliver regeneration-competent cells to the injured tendon, ultimately facilitating its reconstruction and restoring functionality.

Stem cells have been commonly used for this purpose due to their high proliferative and immunomodulatory activities and ability to differentiate into target cell types [7]. Tendon regeneration research has primarily focused on mesenchymal stem cells (MSCs) [8,9,10] and tendon stem/progenitor cells (TSPCs) [6,11,12,13,14]. Furthermore, pluripotent stem cells such as bone marrow stem cells (BM-MSCs) [15], induced pluripotent stem cells (iPSCs) [16], embryonic stem cells (ESCs) [17], and amniotic epithelial stem cells (AECs) [18,19,20] have shown potential in differentiating into tenogenic lineages, thereby promoting effective tendon repair. However, the specific mechanisms guiding tenogenic differentiation are not fully understood, and the optimal stem cell source has yet to be determined. In this direction, concerns have been raised regarding the tendency of BM-MSCs to form ectopic bone or express alkaline phosphatase (ALP) post-implantation in vivo in tendons [21,22]. While TSPCs have shown potential, they have not consistently supported tendon healing in adult models, and their isolation poses risks associated with donor site morbidity [23].

Adipose tissue-derived stem cells (ADSCs) are a prominent subset of MSCs that have garnered significant interest in recent research due to several advantageous characteristics [24,25]. Notably, ADSCs are abundant and can be harvested through minimally invasive liposuction procedures, making them easily accessible [26]. They exhibit low immunogenicity and relatively reduced osteogenic tendencies [27], enhancing their appeal for various therapeutic applications. ADSCs have proven effective in tendon tissue engineering projects [28,29], showing promise as seed cells across multiple scaffolding designs such as 3D woven structures with cellulose nanocrystals and aligned electrospun nanofibers [30], 3D-printed Poly(lactide-co-glycolide) (PLGA) enhanced with collagen fibrils, braided poly(l/d) lactide copolymer filaments [31], and P(LLA-CL)/collagen nanoyarn scaffolds reinforced with silk fibroin [29]. These scaffolds have been explored to enhance tenogenic differentiation through combining physical stimuli like uniaxial tension or alternating magnetic fields.

Although the molecular mechanisms related to the tenogenic differentiation of ADSCs are poorly understood, their stimulation by some specific growth factors, such as transforming growth factor (TGF)-β [32,33,34], growth differentiation factors (GDFs) [35,36,37,38], bone morphogenic protein-12 (BMP-12) [33,34], connective tissue growth factor (CTGF) tenascin-C (TN-C) [39], and other bioactive molecules, seem to promote their differentiation into tendon fibroblasts and improve tendon regeneration. Most studies have evaluated the effect of only one or a few factors simultaneously, but to date, the impact on this cell type on a more complex system, such as conditioned media (CM) or secretome, has not been clarified. However, it is known that using stem cells’ CM promisingly enhances the tendon and ligament healing process, as shown by the available preclinical studies [40]. In this context, protocols for producing a teno-inductive “secretome” derived from the co-culture of AECs with tendon explants have been recently defined [41]. These teno-inductive soluble factors, released into the CM only in response to active communication between fetal tendon explants and stem cells, create a favorable microenvironment for inducing tenogenesis in vitro [18,41].

Despite extensive studies on human ADSCs (hADSCs) [32,42,43,44], rat ADSCs (rADSCs) [45], equine ADSCs (eqADSCs) [46], and rabbit ADSCs (rbADSCs) [27], little is known about the ADSCs of ovine species (oADSCs) [47], which have been increasingly used in preclinical studies [48]. Sheep are a valuable model for orthopedic research due to their significant anatomical and physiological parallels with humans [49,50]. Current research on oADSCs is limited, focusing primarily on their isolation and characterization [51,52]. Developing strategies to control their differentiation into tendon-forming cells remains a significant challenge, necessitating further research to optimize their therapeutic potential for tendon regeneration [53].

**Figure 1:**
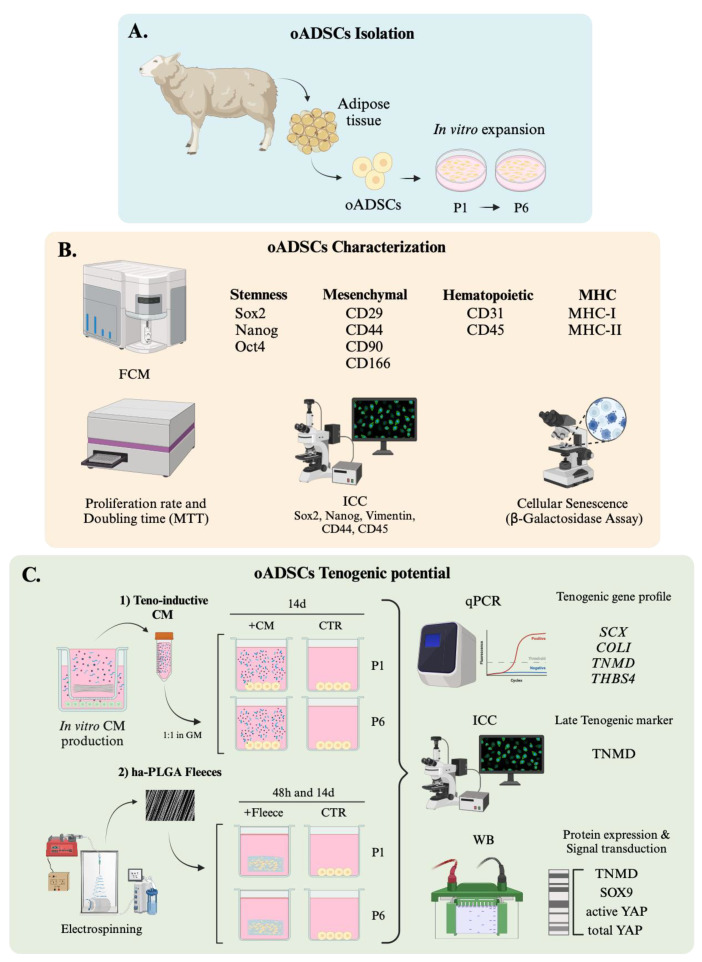
Experimental design. (**A**) oADSCs isolation and expansion until P6. (**B**) oADSCs characterization in terms of the expression of cell surface and intracellular pluripotency markers, self-renewal properties, and morphological features. (**C**) oADSCs’ in vitro tenogenic potential using two validated teno-inductive strategies: (1) exposing oADSCs to teno-inductive CM and (2) engineering oADSCs on ha-PLGA electrospun scaffold to mimic the tendon extracellular matrix with oriented fibers. Morphological, immunocytochemical, and molecular biology investigations were used to define oADSCs biological response.

Starting from these premises, this study aimed to characterize oADSCs during in vitro expansion by studying their morphological features, self-renewal properties, as well as the expression of cell surface markers for MSCs [54] and intracellular pluripotency markers. Once this essential preliminary information to standardize the expansion of stem cells during the pre-transplantation phase was collected, the study then explored the in vitro tenogenic potential of oADSCs using two validated teno-induction strategies: (a) exposing stem cells to teno-inductive CM-containing soluble factors derived from fetal tendon explants [41], and (b) engineering them on a tendon mimetic electrospun scaffold with highly aligned PLGA (ha-PLGA) fibers to mimic the tendon extracellular matrix with oriented fibers [54,55,56,57,58,59]. Morphological, immunocytochemical, and molecular biology investigations were performed on oADSCs under different culture conditions to define their biological response and to evaluate their potential use in veterinary regenerative medicine to cure tendinopathies (Figure 1).

## 2. Materials and Methods

### 2.1. Ethic Statements

No ethical statement was required for this study since the biological samples were collected from ovine animals (*Ovis aries*) slaughtered for food purposes.

In particular, this research utilized materials obtained from a commercial slaughterhouse, where animals are processed as part of standard industry practices. The acquisition of these materials did not involve any additional procedures or interventions beyond those routinely performed in the slaughterhouse. Consequently, ethical approval specific to the study was not required.

### 2.2. oADSCs Isolation and Culture Amplification

oADSCs from ovine tail adipose tissue were isolated from three female donors (n = 3 animals) of around 18 to 24 months old in collaboration with Fat-Stem laboratories (http://www.fat-stem.com) (Figure 1A). The cells were isolated using a kit provided by Fat-Stem, and a nondisclosure agreement (NDA) protects the details of the isolation procedure. The freshly isolated oADSCs (P_0_) from each animal were cultured at 3000 cells/cm^2^ density in a growth medium (GM) consisting of α-Minimum Essential Medium Eagle (α-MEM) supplemented with 10% fetal bovine serum (FBS), 1% ultra-glutamine, 1% amphotericin, and 1% penicillin/streptomycin. The cultures were maintained at 38.5 °C in an atmosphere containing 5% CO_2_. Once the cells reached 70% confluence, cells were detached using 0.05% Trypsin-EDTA and expanded under the same conditions from the first (P1) to the sixth (P6) passage.

### 2.3. Flow Cytometry Investigation for oADSCs Characterization

According to our previous report [18], the characterization of oADSCs (Figure 1B) was conducted through flow cytometry, evaluating intracellular stem cell markers (Sox2, Oct4, and Nanog), cell surface mesenchymal markers (CD29, CD44, CD90, CD105, and CD166), hematopoietic markers (CD31, CD34, and CD45), and Major Histocompatibility Complex markers, Class I and II (MHC-I and MHC-II). The primary antibodies used in these analyses were employed as detailed in Table 1. For the flow cytometry staining procedure, approximately 1 × 10^6^ cells per sample were first treated with 100 µL of 20 mM ethylenediaminetetraacetic acid (EDTA) at 37 °C for 10 min to promote cell separation. Subsequently, the cells were washed with 3 mL of a washing buffer consisting of PBS, 0.1% sodium azide, and 0.5% bovine serum albumin (BSA), followed by centrifugation at 400× *g* for 8 min at 4 °C.

For the staining of surface antigens, the cells were resuspended in 100 µL of the same washing buffer containing the appropriate amount of surface antibody and incubated for 30 min at 4 °C in the dark. Afterward, the cells were washed and centrifuged using the conditions stated previously. Intracellular antigen staining involved resuspending the cells in 1 mL of FACS Lysing solution (BD Biosciences, Allschwil, Switzerland), vortexing, and incubating at room temperature (RT) in the dark for 10 min. Following a wash and the centrifugal steps, the cells were permeabilized with 1 mL of Perm 2 solution (BD Biosciences) for another 10 min at RT in the dark. After another washing step, the cells were incubated with the intracellular antibodies for 30 min at 4 °C in the dark. After all staining procedures, the cells were centrifuged (4 °C, 400× *g*, 8 min), fixed in 0.5% paraformaldehyde, incubated for 5 min at RT, washed, and centrifuged again. They were stored at 4 °C in the dark until analysis. The flow cytometry analysis was performed using a FACSCanto instrument (BD Biosciences) with Diva™ software, v9.0 utilizing Rainbow Calibration Particles (6 peaks) and CaliBRITE beads (both from BD Biosciences) for quality control. Debris were excluded from the analysis by gating on morphological parameters using a lymphocyte gate, and 20,000 non-debris events were recorded for each sample.

Data were analyzed using FlowJo™ software v10.6.1 (TreeStar, Ashland, OR, USA), with the mean fluorescence intensity ratio (MFI ratio) calculated by dividing the MFI of positive events by that of adverse events for each marker. Optimal photomultiplier (PMT) gains for each channel were established based on antibody titration under assay conditions, and each sample was analyzed in triplicate to ensure accuracy and reproducibility [60,61].

### 2.4. Cell Proliferation Assay

The proliferative activity of oADSCs (Figure 1B) was assessed using an MTT (3-(4,5-dimethylthiazol-2-yl)-2,5-diphenyltetrazolium bromide) assay (M5655-1G, Sigma, St. Louis, MO, USA), a colorimetric test to determine cell metabolic activity, according to protocols described in previous studies [62,63]. This assay evaluates cell growth through measuring the enzymatic reduction in MTT to formazan by viable cells, which indicates cell viability and proliferation. For this purpose, oADSCs were seeded at a density of (0.3 × 10^5^ cells/well) in 96-well plates. At 24, 48, and 72 h of culture, the medium was removed from each well, and the cells were rinsed with GM. Subsequently, 20 µL of MTT solution (5 mg/mL in PBS) was added to each well. The plates were then incubated at 38.5 °C for 3.5 h, allowing the living cells to reduce the MTT to formazan. After the incubation, the formed formazan crystals were dissolved with 100 μL of dimethyl sulfoxide (DMSO). The absorbance of the dissolved formazan was measured at 595 nm using an EnSpire^®^ Multimode Plate Reader (PerkinElmer, Waltham, MA, USA).

### 2.5. Doubling Time

The growth kinetics in the long-term culture, from P1 to P6, were monitored by counting cell numbers in subsequent passages using a hemocytometer and trypan blue (Figure 1B). To this end, 25,000 cells/mL were seeded in triplicate on 35 mm Petri dishes, cultured at 38.5 °C with 5% CO_2_ until reaching 70% of confluence, and then detached with 0.05% Trypsin-EDTA. Cells that had reached 12 h of culture were considered as the starting point (T_0_). The doubling time was then calculated according to the following formula [64]:

Doubling Time = (Duration × ln(2)/(ln(Final Concentration) − ln(Initial Concentration)))(1)

### 2.6. Senescence-Associated β-Galactosidase Assay

Senescent β-galactosidase activity, a hallmark of cellular senescence, was measured in oADSCs at P1 and P6 using the Senescence Cells Histochemical Staining Kit (Sigma-Aldrich) following the manufacturer’s instructions. For this assay, cells from both passages were plated at 25,000 cells/mL on 35 mm Petri dishes and cultured for 48 h. Post-culture, the cells were rinsed with PBS and fixed with 1.5 mL of 1× Fixing Buffer for 5 min at RT. After another PBS wash, 1 mL of Staining Mixture, prepared according to the manufacturer’s guidelines, was added to each dish. To develop the color, the dishes were incubated overnight at 37 °C without CO_2_. Senescent cells were counted, identifiable by their blue staining under an inverted phase contrast microscope (Leica). The results were expressed as blue-stained (β-galactosidase positive) cells relative to the total cell count. The data were obtained as a percentage and represented as mean ± SD. This assay was conducted in triplicate for each biological sample.

### 2.7. Immunocytochemistry (ICC)

The immunocytochemical analysis (Figure 1B) was performed on oADSCs at P1 and P6 and under different culture conditions to evaluate the following:

- Sox2 and Nanog proteins nuclear localization to determine stem cell characteristics [13].
- Vimentin and CD44 mesenchymal markers expression to assess mesenchymal lineage [4].
- TNMD protein expression to evaluate a late tenogenic differentiation phenotype [45].

Briefly, oADSCs’ samples were fixed in 4% paraformaldehyde for 10 min. After 3 washes with PBS, the samples were permeabilized with 0.01% Triton-X in PBS (5 min at RT). Non-specific bindings were blocked by incubating cells at RT in PBS/1% BSA for 1 h. Primary antibodies for specific binding antigens reported in Table 2 were diluted in 1% (*w*/*v*) BSA/PBS and incubated overnight at 4 °C. Anti-rabbit Alexa Fluor 488-conjugated, anti-mouse Alexa Fluor 488-conjugated, and anti-rat CY3-conjugated secondary antibodies, as shown in Table 2, were used for 1 h at RT for antigen detection. Nuclei were counterstained using 4′,6-diamidino-2-phenylindole (DAPI, VECTASTAIN) at a final dilution of 1:5000 in PBS. Negative controls were prepared by omitting primary antibodies to assess non-specific staining.

The morphometric evaluation of the images was obtained using an Axioskop 2 Plus incident light fluorescence microscope (Carl Zeiss, Oberkochen, Germany) equipped with a CCD camera (Axiovision Cam, Carl Zeiss) with a resolution of 1300 × 1030 pixels; these images were then configured for fluorescence microscopy, and interfaced to a computer workstation, provided with an interactive and automatic image analyzer (Axiovision, Carl Zeiss) [55]. The quantification of the number of cells expressing target-analyzed proteins was determined by counting positive cells/100 cells counterstained with DAPI and then expressing them as a percentage of positive cells. The reaction was performed in triplicate (n = 3) for each experimental condition on each biological replicate (n = 6 animals).

### 2.8. Tenogenic Differentiation

The in vitro teno-differentiative potential of oADSCs was evaluated using two distinct teno-inductive strategies (Figure 1C).

#### 2.8.1. oADSCs Culture with Teno-Inductive CM

oADSCs at P1 and P6 were cultured in SM until reaching 70% of confluence (Figure 1(C1)). Subsequently, the cells were cultured in a teno-inductive CM 1:1 diluted with GM, according to previously established protocols [41]. The teno-inductive CM was obtained from a co-culture of oAECs with fetal tendon explants and collected according to our previous reports [18,41]. oADSCs were incubated in CM for 14 days at 38.5 °C in a 5% CO_2_ atmosphere, and the medium was refreshed every 48 h up to the end of the culture. As the internal positive control (CTR int), the oAECs were used to assess the teno-inductive potential of the CM [41]. Cell morphology was monitored throughout the culture period.

#### 2.8.2. oADSCs Culture on ha-PLGA Fleeces

oADSCs were seeded onto validated highly aligned PLGA (ha-PLGA, PLG8523) fleeces [54,55,65] at 50,000 cells/fleece density (Figure 1(C2)). Before cell seeding, the ha-PLGA fleeces were cut into rectangular pieces of 15 mm × 7 mm each, sterilized with 70% ethanol (EtOH) in 0.9% NaCl/distilled water (di-H_2_O) solution [55], and pre-incubated in SM for 2 h at 38.5 °C and 5% CO_2_. Afterward, oADSCs were engineered on ha-PLGA fleeces, cultured in GM, and incubated for 48 h and 14 days at 38.5 °C with 5% CO_2_. oAECs cultured on ha-PLGA fleeces for up to 14 days served as an internal positive control (CTR int), showcasing their capacity to differentiate under conditions similar to those previously demonstrated [55].

For both teno-inductive conditions, the cells were subjected to morphometric assessment, immunocytochemical staining, and molecular biology (RT-qPCR and Western blot) analyses to evaluate and characterize their differentiative ability.

### 2.9. RT-qPCR Analyses

According to previous reports, the total RNA from CTR, CM-treated, and ha-PLGA fleeces-engineered oADSCs was extracted with TriReagent (Sigma) [41,62,63,66]. The RNA concentrations were quantified using a Thermo Scientific NanoDrop 2000c UV-Vis spectrophotometer at 260 nm. For cDNA synthesis, 1 μg of total RNA from each sample was reverse transcribed using Random Hexamer Primers and Tetro Reverse Transcriptase (Bioline, Luckenwalde, Germany) in a final volume of 20 μL. Real-time quantitative PCR (qPCR) was conducted to evaluate the expression of tenogenic gene markers Scleraxis (SCX), Collagen type I (COL1A1), Tenomodulin (TNMD), and Thrombospondin 4 (THSB4) [41]. The qPCR reactions used the SensiFAST™ SYBR Lo-ROX kit (Bioline); the primer sequences are listed in Table 3. Amplification was performed on a 7500 Fast Real-Time PCR System (Life Technologies, Waltham, MA, USA) using a two-step cycling protocol: 40 cycles at 95 °C for 10 s for denaturation and 60 °C for 30 s for annealing/extension, followed by a melt curve analysis using 7500 Software v2.3 [41]. Each gene was tested in triplicate, and expression levels were normalized to the endogenous reference gene GAPDH. Relative expression levels were calculated using the comparative Ct (ΔΔCt) method, and the results were expressed as fold changes relative to the untreated control samples (CTR) using the formula 2^−ΔΔCt^ [67].

### 2.10. Total Protein Extraction and Western Blot

Western blot analysis was performed on CTR, CM-treated, and ha-PLGA fleeces-engineered oADSCs samples to assess the late tendon marker TNMD [41,54,68,69] and the transcriptional regulators SRY-box 9 (SOX9) [70] and Yes-associated protein (YAP), both involved in stem cell fate and differentiation [71,72,73]. The total proteins were extracted using lysis buffer (50 mMTris HCl pH 8, 250 mMNaCl, 5 mM MEDTA, 0.1% Triton X-100 10%) supplemented with Phosphatase Inhibitor (39055; SERVA Electrophoresis GmbH, Heidelberg, Germany) and Protease Inhibitor Cocktails (P2714; Sigma-Aldrich, St. Louis, MO, USA) diluted according to the manufacturer’s instructions. The cell lysates were incubated on ice for 30 min, followed by centrifugation at 12,000× *g* for 10 min at 4 °C. The protein concentration was then determined using 5 µL of sample and the Quick Start™ Bradford 1x Dye Reagent (Bio-Rad Laboratories, Milan, Italy). Subsequently, 30 µg of total protein from each sample was loaded onto a precast gel with a density gradient of 4–15% (mini-PROTEAN precast gel, Bio-Rad Laboratories, Milan, Italy) and separated in 1X TGS Running Buffer (Bio-Rad Laboratories, Milan, Italy). Proteins were then transferred onto nitrocellulose membranes (1620145; Bio-Rad Laboratories, Milan, Italy) using Trans-Blot Turbo 5X Transfer Buffer (10026938; Bio-Rad Laboratories, Milan, Italy) and Trans-Blot Turbo Transfer (Bio-Rad Laboratories, Milan, Italy). The membranes were blocked with Every blot-blocking solution (Bio-Rad Laboratories, Milan, Italy) for 5 min at RT. Primary antibodies against TNMD, SOX9, active YAP (a-YAP) and total YAP (YAP), and α-Tubulin were diluted in 1X TBS 1% Casein Blocker (Bio-Rad Laboratories, Milan, Italy), as described in Table 4, and incubated overnight at 4 °C. Specific secondary HRP-conjugated antibodies were diluted in the previously mentioned solution and incubated for 1 h at RT. The detection of target proteins was performed using ClarityMax ECL reagent (Bio-Rad Laboratories, Milan, Italy), and chemiluminescent signals were captured with the ChemiDoc MP Imaging System. The densitometric analysis of the blots was conducted using ImageJ (ImageJ 1.53k, NIH, Bethesda, MD, USA; https://imagej.net/). The relative protein expression levels of TNMD and SOX9 were normalized to the corresponding α-Tubulin levels in each sample. In the case of the YAP protein, the a-YAP expression levels were first normalized to the corresponding total YAP expression and then with α-Tubulin levels.

### 2.11. Statistical Analysis

The quantitative data presented in this manuscript were obtained by analyzing at least three independent samples for each experimental condition (n = 3), using three biological replicates (n = 3 animals). The Shapiro–Wilk test was utilized to assess the normality of the data distributions. The values are expressed as the mean ± SD, and the differences among the groups were evaluated using one-way ANOVA with Tukey’s post hoc test, performed using GraphPad Prism 10 (GraphPad Software, San Diego, CA, USA). A significance level was set at *p* < 0.05.

## 3. Results

### 3.1. oADSCs Maintained the Expression of Stemness and Mesenchymal Markers during In Vitro Amplification

**Figure 2:**
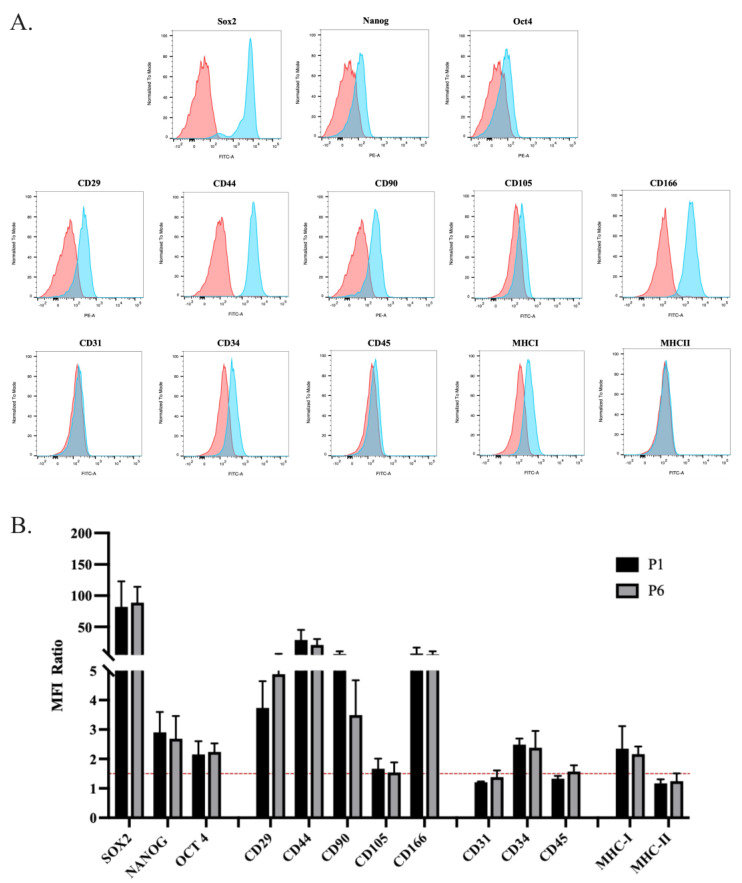
Cytofluorimetric characterization of oADSCs at P1 and P6 of culture in GM. (**A**) Graphical representations of the flow cytometry analyses conducted on oADSCs at P1 regarding stemness, mesenchymal (adhesion molecules), hematopoietic, and MHC markers. Histograms in blue represent stained cells, while the red ones represent the control of the un-stained group. (**B**) Histogram representation of the analyzed markers within oADSCs (P1 and P6) expressed as the mean fluorescence intensity (MFI) ratio. The red line represents the positivity threshold. Data were obtained as means ± SD values of 3 replicates for each sample.

The cells were cultured up to P6 and characterized at P1 and P6 using flow cytometry in terms of the expression of MSCs markers. The antigen expression profile in the populations under examination has mainly given unimodal patterns (Figure 2A), in which the entire population is expressing or not expressing. The entire Gaussian curve represents the population shifts, indicating the totality of the population (100%). In detail, the oADSCs at P1 displayed the expression of the stemness markers Sox2, Nanog, and Oct4, along with the surface adhesion molecules CD29 and CD166 and the mesenchymal markers CD44 and CD90, and the CD34 hematopoietic marker (Figure 2A). In the case of Sox2 expression, it can be seen that the positivity is expressed in a bimodal way, with cells showing a lower peak or a higher peak relative to the antigen, possibly related to the translocation of transcription factors. The cells were negative for the hematopoietic markers CD31 and CD45 (Figure 2A) and the major histocompatibility complex antigen MHC-II. Additionally, there was a low expression of CD105 and MHC-I (Figure 2A). The antigenic profile observed at P1 was consistently maintained at P6. The statistical analysis revealed no significant differences in the expression levels of these markers between P1 and P6 oADSCs (Figure 2B, *p* > 0.05), suggesting that the phenotypic characteristics of the cells remained stable under the used-culture conditions.

### 3.2. oADSCs Maintained an Unaltered Proliferation Rate and Metabolic Status during Amplification In Vitro

**Figure 3:**
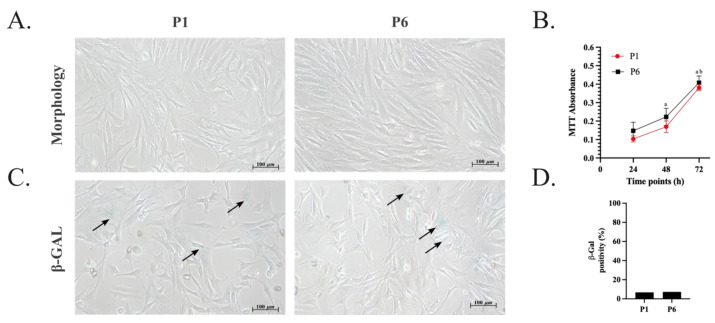
Morphology, proliferation rate, and metabolic activity of oADSCs at P1 and P6 during amplification in GM. (**A**) Representative phase-contrast images of oADSCs from P1 and P6 after 72 h of culture show fibroblast-like morphology. Scale bar of 100 µm. (**B**) The proliferation rate of oADSCs at P1 and P6 cultured for 24 h, 48 h, and 72 h was measured through MTT colorimetric assay and represented as absorbance mean values ± SD. (a vs. P1/P6 24 h, b vs. P1/P6 48 h, *p* < 0.05). The data for each biological sample were obtained in triplicate. (**C**) Metabolic activity assessment of oADSCs from P1 and P6 cultured for 48 h and observed through an inverted phase contrast microscope after β-GAL staining (blue). Scale bar of 100 µm. The black arrows indicate β-GAL positive cells. (**D**) The expression of β-GAL senescence assay was calculated by counting β-GAL positive cells/100 total cells obtained from three replicates for each biological sample.

The amplified oADSCs showed plastic-adherent fibroblast-like morphology during the days of culture (Figure 3A). Moreover, these cells underwent in vitro amplification, and the doubling time for each passage from P1 to P6 was further calculated. On average, 5–7 days were required for the cells to reach 70% confluence at each passage. Specifically, the mean cell doubling time during passages P1 and P2 was approximately 24 ± 8 h. Although no statistically significant differences were observed in the doubling time across the passages, a slight decrease in the time required to complete each passage was noted from P3 to P6, with a doubling time of about 19 ± 9 h. Concurrently, an increase in the number of living cells was monitored through the absorbance measured in a colorimetric MTT assay at 24, 48, and 72 h of culture, indicating an increased total metabolic activity and thus supporting cell proliferation over time (Figure 3B, *p* < 0.05). No significant differences in metabolic activity were detected at the same time points across different passages (Figure 3B, *p* > 0.05). In support of these results, little cellular senescence was observed in the oADSCs (Figure 3C). Indeed, about 90% of the total cells were metabolically active in both passages, and an occasional signal of β-galactosidase (β-GAL) activity was detected (Figure 3D). Indeed, only 10% and 11% of the cells at P1 and P6 were positive for β-GAL staining, respectively (Figure 3D, *p* > 0.05).

**Figure 4:**
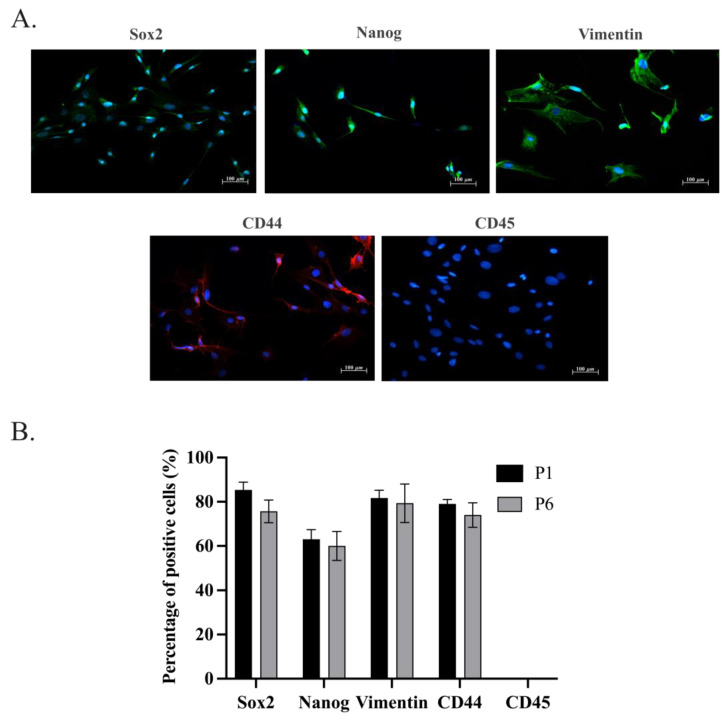
Immunocytochemical characterization of oADSCs at P1 and P6 of culture in GM. (**A**) Immunofluorescence representative images showing the distribution of Sox2 and Nanog intracellular stemness markers (Green fluorescence), Vimentin (Green fluorescence), CD44 (Red fluorescence) mesenchymal markers, and CD45 (Red fluorescence) hematopoietic marker, in oADSCs at P1 after 48 h of culture. Nuclei were counterstained with DAPI (Blue fluorescence). Scale bar of 100 µm. (**B**) Histograms represent the percentage of oADSCs positive cells, at P1 and P6, to the different analyzed markers, expressed as the number of positive cells/100 total cells obtained from three replicates for each biological sample.

Immunocytochemistry investigations revealed a similar expression pattern in the stemness and in the mesenchymal markers analyzed within oADSCs at P1 and P6 (Figure 4; *p* > 0.05). In particular, the oADSCs at P1 after 48 h of culture in GM exhibited the expression for stemness markers Sox2 and Nanog (Figure 4), principally localized in about 88% and 65% of the nuclei of the positive cells, respectively (Figure 4B). Moreover, around 80% of the cells expressed Vimentin and CD44 mesenchymal marker proteins (Figure 4). No cells showed positivity to the expression of the hematopoietic marker CD45 (Figure 4). The same IHC results were also obtained in the oADSCs at P6 of amplification. These data confirm the mesenchymal lineage of the oADSCs and support the phenotypic stability over the culture period highlighted by flow cytometry analyses (Figure 2).

### 3.3. oADSCs Tenogenic Potential Is Strictly Dependent on the In Vitro Differentiation Strategy

**Figure 5:**
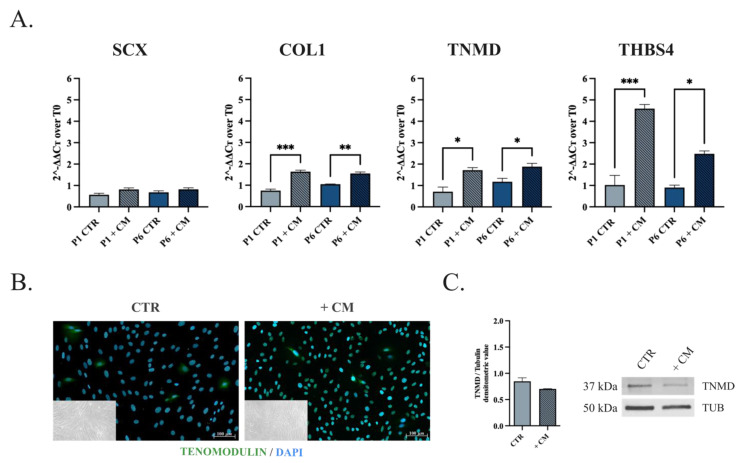
Assessment of the tenogenic differentiation of oADSCs after culture with teno-inductive CM. (**A**) Quantitative RT-PCR evaluation of SCX, COL1, TNMD, and THBS4 tenogenic markers mRNA expression on oADSCs from P1 and P6 untreated (CTR) and treated with CM (+CM) for 14 days. (**B**) Representative IHC assessment of TNMD protein expression (Green fluorescence) and phase-contrast images (in the inner box) of oADSCs at P1 untreated and treated with teno-inductive CM for 14 days of culture. Nuclei were counterstained with DAPI (Blue fluorescence). Scale bar of 100 µm. (**C**) Representative Western blot image of α-TNMD proteins expression on oADSCs at P1 untreated (CTR), treated with CM (+CM) for 14 days, and relative densitometric values normalized on α-TUBULIN expression. Data were expressed as means ± S.D. of three replicates/each biological sample. *, **, *** Statistically significant values between the different studied groups (*p* < 0.05, *p* < 0.01, and *p* < 0.001, respectively).

**Figure 6:**
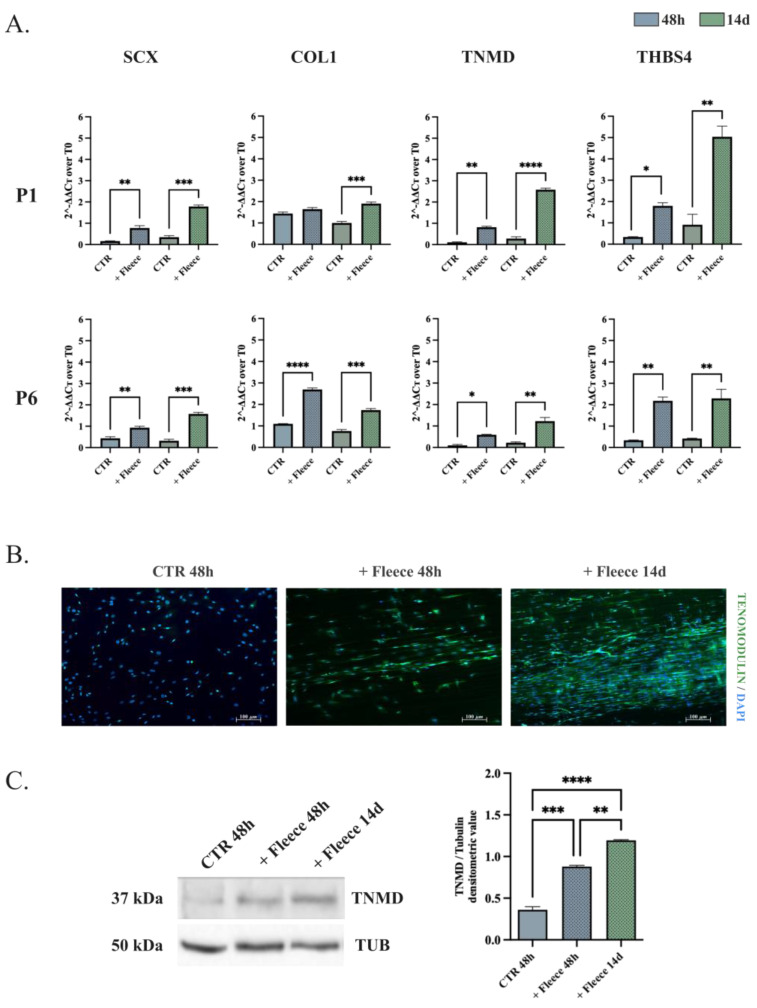
Assessment of the tenogenic differentiation of oADSCs at P1 after being cultured on ha-PLGA fleeces. (**A**) Quantitative RT-PCR evaluation of SCX, COL1, TNMD, and THBS4 tenogenic markers mRNA expression on oADSCs at P1 and P6, cultured on Petri dish (CTR) or ha-PLGA fleeces (+Fleece) for 48 h and 14 days. (**B**) Immunocytochemical assessment of TNMD protein expression (Green fluorescence) within oADSCs at P1 cultured on a Petri dish for 48 h (CTR 48 h) and on ha-PLGA fleeces for 48 h and 14 days (+Fleece 48 h and +Fleece 14 d, respectively). Nuclei were counterstained with DAPI (Blue fluorescence). Scale bar of 100 µm. (**C**) Representative Western blot images for α-TNMD protein expressions on oADSCs at P1 in untreated (CTR 48 h) and engineered on ha-PLGA fleeces for 48 h (+Fleeces 48 h) and 14 days (+Fleeces 14 d), and relative densitometric values normalized on α-TUBULIN expression. Data were expressed as means ± S.D. of three replicates/each biological sample. *, **, ***, **** Statistically significant values between the different studied groups (*p* < 0.05, *p* < 0.01, *p* < 0.001 and *p* < 0.0001, respectively).

The oADSCs differentiation capacity toward the tenogenic lineage was carried out in vitro using two stimuli. Cells at P1 and P6 were exposed to teno-inductive CM for 14 days (Figure 5) or engineered on ha-PLGA fleece for 48 h and 14 days in GM (Figure 6). The differentiation process was analyzed in oADSCs untreated (CTR), CM-treated (+CM), or engineered on ha-PLGA fleeces (+Fleece) by combining Real-Time PCR, Western blot, and Immunofluorescence analyses (Figure 5 and Figure 6).

The RT-qPCR results indicated that the oADSCs at P1 and P6 cultured in GM (CTR) for 14 days expressed the basal levels of early SCX, mature COL1, late TNMD, and THBS4 tenogenic markers gene expression (Figure 5A) that remained at similar levels in the different passages of culture (*p* > 0.05). When oADSCs were cultured under a teno-inductive CM stimulus, the upregulation of the tendon-related markers COL1, TNMD, and THBS4 was observed in treated cells compared to their CTR (*p* < 0.05, Figure 5A), without any modulation of SCX (*p* > 0.05, Figure 5A). However, despite tendon-related genes being upregulated, oADSCs failed to acquire the tenogenic phenotype fully. Indeed, independently of the culture passage, no significant morphological changes have been observed within CM-treated oADSCs at the end of the culture period. Indeed, they maintained their native fibroblast-like morphology (Figure 5B, phase contrast images in the inner box), with few cells expressing weak cytoplasm positivity to the TNMD protein tested as a late tenogenic marker (Figure 5B). As a confirmation, the TNMD protein quantification in CM-treated oADSCs and CTR showed similar levels (Figure 5C, *p* > 0.05).

Interestingly, oADSCs seeded on ha-PLGA fleeces were able to differentiate towards the tenogenic lineage, showing molecular and morphological signs of differentiation already at 48 h (Figure 6), independently from the passage of culture. The expression profile of the P1 oADSCs cultured on ha-PLGA fleeces for 48 h was upregulated for three out of four tendon-related genes (SCX, TNMD, and THBS4), while overall there was an overexpressed result (SCX, COL1, TNMD, and THBS4) in the P6 expanded ones (*p* < 0.05; Figure 6A). The upregulation of tendon-related transcripts was simultaneously accompanied by morphological cell changes and late tendon-related glycoprotein (TNMD) accumulation. Indeed, starting from 48 h, oADSCs cultured on ha-PLGA fleeces aligned and began to elongate along the longitudinal axis of the fibers, acquiring a tenocyte-like morphology and predominantly expressed transmembrane TNMD protein, which increased in expression up to day 14 of culture (Figure 6B). Western blot analysis supported this teno-differentiative response on oADSCs at P1 (Figure 6C). Notably, the TNMD synthesis was significantly increased in oADSCs cultured on ha-PLGA fleeces already after 48 h (Figure 6C, *p* < 0.01 vs. CTR), until doubling its content after 14 days (Figure 6C, *p* < 0.001 vs. CTR).

Overall, the results summarized in Figure 5 and Figure 6 indicate that oADSCs’ tenogenic plasticity highly depends on the differentiation in vitro strategy employed during their culture.

### 3.4. SOX9 Downregulation Leads to In Vitro Tenogenic Differentiation of oADSCs in a Teno-Inductive Strategy-Dependent Manner

**Figure 7:**
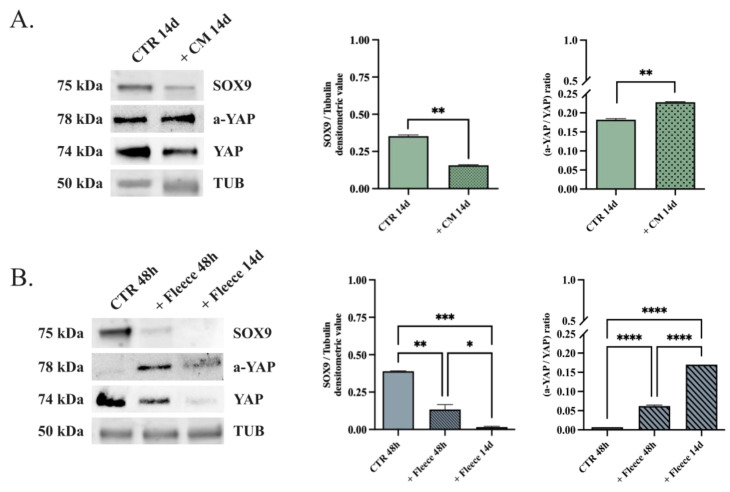
SOX9 and YAP protein expression assessment on oADSCs cultured under both teno-differentiative conditions. (**A**) Representative Western blot images for SOX9, a-YAP, and total YAP protein expressions in oADSCs at P1 cultured in GM on a Petri dish for 14 days (CTR 14 d) and treated with teno-inductive CM for 14 days (+CM 14 d) (**B**) Representative Western blot images of SOX9, a-YAP, and YAP protein expression in oADSCs at P1 cultured in GM on a Petri dish for 48 h (CTR 48 h) or seeded on ha-PLGA fleeces for 48 h and 14 days (+Fleece 48 h and +Fleece 14 d, respectively). Relative densitometric values of SOX9, a-YAP and total YAP were normalized on α-TUBULIN expression. Data were expressed as means ± S.D. of three replicates/each biological sample. *, **, ***, **** Statistically significant values between the different studied groups (*p* < 0.05, *p* < 0.01, *p* < 0.001 and *p* < 0.0001, respectively).

Based on the data obtained, the inverse role of SOX9 and YAP transcriptional factors within oADSCs differentiation was assessed to investigate the mechanisms underlying in vitro tenogenesis in depth (Figure 7).

The results indicated that oADSCs at P1 expressed high levels of SOX9 protein (Figure 7; oADSCs CTR 48 h and 14 days). Both teno-differentiative conditions lead to the downregulation of SOX9, even if this effect was strictly dependent on the differentiation strategy (Figure 7). Notably, the oADSCs exposed to CM significantly halved the SOX9 protein levels after 14 days (Figure 7A; *p* < 0.001 vs. CTR 14 days). This level of protein downregulation was reached in cells engineered on ha-PLGA fleeces after 48 h (fleece 48 h, *p* < 0.001 vs. 48 h CTR). Instead, the engineered oADSCs on PLGA fleeces reached undetectable SOX9 levels in 14 days (Figure 7B; *p* < 0.0001 vs. 48 h CTR and *p* < 0.05 vs. fleece 48 h).

An opposite behavior was recorded for a-YAP. Indeed, it progressively increased during the process of tendon differentiation, albeit again in a manner strictly dependent on the differentiation procedure used (*p* < 0.01 vs. CTRs). Indeed, the YAP expression ratio (a-YAP/YAP) in oADSCs exposed to CM (Figure 7A) increased by approximately 1.2-fold after 14 days of culture (Figure 7B). YAP’s activation response was more evident in cells engineered on ha-PLGA fleeces. The ratio a-YAP/YAP increased by 9-fold and 24-fold after 48 h and 14 days of culture, respectively. Similar results were also obtained in the oADSCs at P6. These findings confirmed the role of the mechano-sensing pathway in the tendon differentiation of oADSCs and its more effective influence after the ha-PLGA-mediated tissue-engineered strategy. In addition, the inverse influence of SOX9 downregulation and YAP activation in tendon differentiation was confirmed by the oADSCs.

## 4. Discussion

This study demonstrates, for the first time in our knowledge, that oADSCs have a great self-renewal property by preserving their mesenchymal and stemness phenotype during amplification without impairing their propensity to differentiate in a tenogenic lineage. The oADSCs characterization profile aligns with the literature data [51,74,75]. Considering that the reported range of cell passages for this purpose extends up to P7 [76], this research focuses on oADSCs that are expanded until P6, since higher passages are associated with cellular senescence and subsequent changes in biological properties [64,75]. This restricts the potential use of ADSCs in regenerative medicine and clinical applications [64,75]. When cultured under standard conditions, oADSCs exhibited plastic adherence and a fibroblast-like morphology, maintaining high proliferative capacity up to P6 without signs of cellular aging. oADSCs consistently expressed mesenchymal-related antigens CD29, CD166, CD44, CD90, and CD34 throughout the culture passages and showed a low expression of CD105 and MHC-I. They tested negative for the hematopoietic-related markers CD31 and CD45 and MHC-II. This low expression of MHC I and II in the oADSCs makes them ideal candidates for allotransplantation, considering the importance of sheep models as large mammals for preclinical studies [77,78,79]. The CD34 positivity of oADSCs is in agreement with the literature data, demonstrating that CD34 is expressed by a multitude of nonhematopoietic cell types including MSCs, muscle satellite cells, corneal keratocytes, interstitial cells, epithelial progenitors, and vascular endothelial progenitors [80,81]. The ADSCs research, being predominantly carried out using culture-expanded cells, has led to a recent acceptance of CD34 as a marker for isolated ADSCs. Thus, there remains interesting aspects of CD34 biology to be explored and understood [81]. Furthermore, the cells were positive for the stemness-related markers Oct4, Nanog, and Sox2, with the latter being predominantly localized in most cells’ nuclei. The insights gained from this study are crucial for advancing our understanding of the ovine stem cell source, thus opening greater awareness of the use of ADSC. In particular, oADSCs could be used in preclinical settings to collect information about their potential applications in tendon stem cell-based therapy. This is especially relevant when considering the recognized value of the ovine model as a tendon translational model due to its anatomical and physiological similarities to humans [82,83,84,85]. Furthermore, after confirming the stable phenotype and stemness profile from P1 to P6, the tenogenic differentiation potential of oADSCs was assessed according to the functional recommendations provided by the International Federation for Adipose Therapeutics and Science (IFATS) [75].

Although regenerative medicine has made significant advances, the mechanisms of tenogenesis and robust in vitro tendon differentiation protocols are yet to be fully understood. This is mainly due to the absence of standardized methods and an optimal panel of markers for characterizing the stages of tenogenic differentiation [6]. Validating teno-inductive techniques is crucial for in vitro models, as they are a fundamental precursor to any application involving differentiated cells. Various teno-inductive approaches are proposed for inducing tenogenic differentiation from different stem cell sources, including ADSCs. These methods include using single or multiple specific growth factors and/or biomimetic materials and bioreactors. However, these techniques have yet to be proven reliable in consistently inducing a fully committed tendon phenotype. The protocols adopted in the present research have been selected from previously validated sources of ovine stem cells [18,41,54,55,68]. Implementing this alongside the in vitro differentiation strategy, it was also demonstrated that oADSCs can be directed toward the tenogenic lineage at both genotypic and phenotypic levels, even after P6. To our knowledge, none of the literature data has reported on ovine ADSCs’ differentiative capacity towards the tenogenic lineage in contrast to their well-documented abilities for chondrogenic, adipogenic, and osteogenic plasticity [51,86,87,88,89]. Of note is that the tendon-related to the in vitro plasticity of oADSCs strictly depended on the in vitro differentiation protocols, thus adding new advances for the practical use of these stem sources in future regenerative medicine protocols. Indeed, oADSCs appear to move towards tendon differentiation more effectively when engineered on ha-PLGA fleeces, thus demonstrating their greater propensity to respond to the physical/chemical stimuli promoted during cell adhesion on a scaffold surface mimicking the tendon ECM, rather than to tendon-inductive soluble factors driving fetal tendon development [18,41].

The exposure of oADSCs to CM did not induce a complete tenogenic commitment even after 14 days. This tendon differentiation protocol exclusively induced the upregulation of late tenogenic markers (TNMD, COLI, and THSB4) while not influencing SCX transcripts. Several pieces of evidence confirmed the crucial role of SCX, either during fetal development for neotendon formation or during the post-natal lifetime, in tissue maturation and repair. Indeed, this transcription factor also maintains a vital role in the homeostasis of adult tendons as a co-activator of other tendon-related genes, such as COL1 and TNMD [90,91]. Its concomitant activation with TNMD is, for example, required to create collagen networks and tendon extracellular matrix organization properly [59,62,92]. TNMD is also considered a late pivotal marker of mature tenocytes in vitro and in vivo, orchestrating the formation of collagen fibrils [91,93,94]. Moreover, THBS4 contributes to the regulation of ECM deposition and in the repair of myotendinous junctions (MTJs) [95,96]. Thus, the increase in SCX downstream effectors (TNMD, THBS4, and COL1) recorded on oADSCs after 14 days of exposure to CM treatment may suggest an earlier SCX activation. However, it can be assumed that the degree, as well as the timing of SCX activation induced by CMs, are not adequate to promote the oADSCs’ terminal differentiation toward the tenogenic lineage, as confirmed by the low level of accumulation of the transmembrane glycoprotein TNMD and by the failure of the stem cells in changing their shape. Overall, these data suggest that the exposure of oADSCs to CM, besides their induction of late tendon-related genes, was insufficient in leading the cells toward a final tendon commitment. This effect could be a result of the lack of the adequate concentration/composition of soluble factors that did not coordinate in oADSCs, which are the complex multiple biological mechanisms needed for tenogenesis, as previously demonstrated in oAECs [41]. This evidence was previously recorded in other stem cell sources where diverse biological responses were recorded in response to the same tendon inductive molecule [6]. This incomplete tenogenic effect observed in CM-treated oADSCs could be due to different intracellular temporal translational mechanisms highlighting the complex interplay of biochemical factors and the cellular environment in in vitro differentiation. Accordingly, it has been evidenced that the supplementation with TGF-β3 in a medium can significantly regulate the temporal expression of tenogenic markers in both ADSCs and BM-MSCs cultures [97].

Moreover, it cannot be ruled out that oADSCs can be partially pre-committed towards other cell lineages [98], which may not be reverted by the bioactive factors present in the teno-inductive CM. The persistence of SOX9 expression in oADSCs exposed to CM for 14 days supports this hypothesis. Previous evidence demonstrates that the forced expression of SOX9 alone can induce monolayered TNMD-expressing tenocytes into chondrocyte cells [99]. Furthermore, the persistent overexpression of SOX9 determined the loss of tenogenic specific phenotypes and related gene expression profiles in tenocytes [99]. It is well known that the modulation of SOX9, as with other transcription factors, is essential to promote cell trans-differentiation by inducing specific tissue lineage competence, starting from a primitive common cell population. SOX9 transcription factor, in particular, has to be only temporally expressed in primitive tendons during the early development phase [100,101] since it is critical for musculoskeletal development in driving the recruitment of mesenchymal cells [70] towards chondrocyte and cartilage [102]. The results obtained in the present research demonstrated that the two tendon inductive protocols exerted a diverse ability to inhibit the synthesis of SOX9. Indeed, the transcription factor persists for 14 days in oADSCs exposed to CM treatment while it was switched off entirely in cells exclusively engineered on ha-PLGA fleeces. The presence of SOX9 may represent a molecular interference for completing tenogenic commitment in oADSCs exposed to CM-mediated protocol.

The greater teno-inductive effects promoted in oADSCs engineered on electrospun ha-PLGA fleeces appeared to instead be mediated by the activation of YAP signaling as previously demonstrated in oAECs cultured on these biomimetic constructs, characterized by their highly aligned fiber topology and mechanical properties, closely mimicking the native tendon ECM [54,68]. Similarly to oAECs, oADSCs engineered on ha-PLGA quickly and uniformly adhered to the surface of the fleeces, activating the mechano-sensing response in a time-dependent manner, as demonstrated by the sustained YAP activation. This mechano-sensing mechanism may also be responsible for the negative modulation recorded on SOX9 and, consequently, for the more rapid and effective ability of oADSCs to move toward the tenogenic lineage when cultured on ha-PLGA seeded cells. This latter hypothesis has been demonstrated in other cell models [73,103,104], where SOX9 was confirmed to be a transcription factor downstream of YAP activation in controlling cell plasticity and fate. Certainly, the overexpression profiles of YAP and downregulation of SOX9 were already recorded in oADSCs cultured for 48 h on ha-PLGA fleeces where an advanced tendon-related profile (upregulation of SCX, COL1, TNMD, and THBS4) was observed. The genomic profile was also consistent with the morphological and proteomic modifications observed. In particular, the engineered oADSCs, after 48 h of culture, displayed a tenocyte-like shape, were enriched in TNMD glycoprotein, and were resultingly aligned along the longitudinal axis of the fibers. This teno-inductive effect was more pronounced at 14 days of culture, supporting the persistence of the mechano-sensing oADSCs’ response to the topological cues of the ha-PLGA fleeces. The mechano-sensing tendon differentiation induced by PLGA scaffolds has been previously demonstrated on other typologies of stem cells. Notably, it has been reported that a PLGA scaffold combined with a collagen-fibrin hydrogel supports hADMSCs tenogenic differentiation with a significant effect when combined with TGF-β3 [105]. Similarly, hADSCs exhibited an enhanced expression of tendon-specific genes after being seeded on a Thymosin Beta-4-loaded PLGA/PLA nanofiber/microfiber hybrid yarn-modified device [106]. Specifically, aligned PLGA fibers have been shown to promote tenogenesis by activating the YAP signaling pathway, a crucial mechanotransduction mechanism [107,108]. Previous studies performed on AECs have demonstrated how scaffold topography and topology are able to enhance cells’ teno-differentiation and immunomodulation through the activation of the YAP mechanotransducer, which translocates into the nuclei of the engineered cells [68]. YAP activation is a recognized intracellular pathway for promoting tendon differentiation in MSC, since it inhibits chondrogenic commitment and chondrocyte proliferation [109]; essentially, a reduction in YAP activity has been associated with chondrogenesis in vivo. Additionally, it has been demonstrated that YAP is deactivated during the in vitro chondrogenesis of human MSCs, and the recombinant overexpression of human YAP in murine C3H10T1/2 inhibits chondrogenesis [110]. Similarly, the hyperactivation of endogenous YAP has been reported to impair chondrocyte differentiation and maturation, leading to chondrodysplasia in Mob1a/b-deficient mice. It is also worth noting that in this experiment, the biological effect has been linked to the suppression of SOX9, a key upstream regulator of chondrogenesis [111].

This study provides new insights into the biology of oADSCs, revealing their impressive self-renewal capacity and tenogenic plasticity. The findings confirm that oADSCs can be effectively utilized in preclinical settings, validating stem cell-based tendon regenerative therapies on a highly valuable animal model. The study suggests that tissue engineering strategies are the most effective protocol for exploiting the tenogenic tendency of this stem cell source. The chemical/physical stimuli from highly aligned fiber constructs strongly induce mechanosensory responses in oADSCs, promoting tenogenesis through the favorable modulation of the YAP–SOX9 axis. These results have promising therapeutic implications for enhancing the use of ADSC in the veterinary regenerative medicine field, where this stem cell source has been widely proposed for treating tendinopathies, offering a hopeful outlook for future advancements in this area.

## Figures and Tables

**Table 1:** Summary of antibodies used in cytofluorimetric analysis.

Antigen	Conjugated Fluorescent Probe	Company Details	
Stemness markers			
Sox2	FITC	Abcam	Cambridge, UK
Nanog	PE	Chemicon Int.	Billerica, MA, USA
Oct4	PE	Becton Dickinson	BD, San Jose, CA, USA
Adhesion molecules			
CD29	PE	VMRD	Pullman, WA, USA
CD44	FITC	Becton Dickinson	BD, San Jose, CA
CD90	PE	AbD Serotec	Oxford, UK
CD166	FITC	Ancell	Stillwater, MN, USA
CD105	FITC	Ancell	Stillwater, MN, USA
Hematopoietic markers			
CD31	FITC	AbD Serotec	Oxford, UK
CD34	FITC	AbD Serotec	Oxford, UK
CD45	FITC	AbD Serotec	Oxford, UK
MHC antigens			
Class-I HLA	FITC	Novus Biologicals	Cambridge, UK
Class-II HLA-DR	FITC	Abcam	Cambridge, UK

**Table 2:** Details of primary and secondary antibodies used for immunocytochemistry assay.

Primary Antibody (Company Information)	Primary Antibody Dilution	Secondary Antibody (Company Information)	Secondary Antibody Dilution
Vimentin (Dako M0725)	1:50	Mouse Alexa Fluor 488 (Abcam ab 150113)	1:100
CD45 (DB BIOTECH DB042)	1: 50	Rabbit Alexa Fluor 488 (Abcam ab 150113)	1:100
CD44 (Abcam ab119335)	1:100	RAT CY3-conjugated (Chemicon Ap136c)	1:500
TNMD (Biorbyt orb101154)	1:100	Rabbit Alexa Fluor (Abcam ab 150077)	1:500
Sox2 (Abcam ab69893)	1:200	Rabbit Alexa Fluor (Abcam ab 150077)	1:500
Nanog (Millipore, AB9220)	1:1000	Rabbit Alexa Fluor (Abcam ab 150077)	1:500

**Table 3:** Primer details used for RT-qPCR analysis.

Gene	Forward Primer (5′ to 3′)	Reverse Primer (5′ to 3′)	Product Size (bp)
SCX ^b^	AACAGCGTGAACACGGCTTTC	TTTCTCTGGTTGCTGAGGCAG	299
COL1A1 ^b^	CGTGATCTGCGACGAACTTAA	GTCCAGGAAGTCCAGGTTGT	212
TNMD ^b^	TGGTGAAGACCTTCACTTTCC	TTAAACCCTCCCCAGCATGC	352
THSB4 ^a^	CCGCAGGTCTTTGACCTTCT	CAGGTAACGGAGGATGGCTTT	231
GAPDH ^b^	CCTGCACCACCAACTGCTTG	TTGAGCTCAGGGATGACCTTG	224

Primers used in previous reports: ^a^ [41] ^b^ [68].

**Table 4:** Details of primary and secondary antibodies used for Western blot assay.

Antibodies Used for WB Analysis			
TNMD (Abcam ab203676)	1:500	Anti-rabbit HRP-conjugated. (Santa Cruz, sc-516102)	1:10,000
SOX9 (Abcam ab182579)	1:5000	Anti-rabbit HRP-conjugated. (Santa Cruz, sc-516102)	1:10,000
YAP (Cell Signaling 14074S)	1:500	Anti-rabbit HRP-conjugated. (Santa Cruz, sc-516102)	1:10,000
a-YAP (Cell Signaling 29495S)	1:500	Anti-rabbit HRP-conjugated. (Santa Cruz, sc-516102)	1:10,000
Tubulin (Cell Signaling 3873S)	1:2000	Anti-mouse HRP-conjugated. (Santa Cruz, sc-516102)	1:10,000

## Data Availability

The raw data supporting this article’s conclusions will be made available upon request by the authors without undue reservation.
